# GABA Modulates Frequency-Dependent Plasticity in Humans

**DOI:** 10.1016/j.isci.2020.101657

**Published:** 2020-10-08

**Authors:** Caroline A. Lea-Carnall, Stephen R. Williams, Faezeh Sanaei-Nezhad, Nelson J. Trujillo-Barreto, Marcelo A. Montemurro, Wael El-Deredy, Laura M. Parkes

**Affiliations:** 1 Division of Neuroscience and Experimental Psychology, School of Biological Sciences, Faculty of Biology, Medicine and Health, University of Manchester, Manchester Academic Health Science Centre, Manchester, UK; 2 Division of Informatics, Imaging and Data Science, School of Health Sciences, Faculty of Biology, Medicine and Health, University of Manchester, Manchester Academic Health Science Centre, Manchester, UK; 3 Centro de Investigación y Desarrollo en Ingeniería en Salud, Universidad de Valparaíso, Valparaíso, Chile

**Keywords:** Molecular Neuroscience, Systems Neuroscience, Cognitive Neuroscience

## Abstract

Frequency-dependent reorganization of the primary somatosensory cortex, together with perceptual changes, arises following repetitive sensory stimulation. Here, we investigate the role of GABA in this process. We co-stimulated two finger tips and measured GABA and Glx using magnetic resonance (MR) spectroscopy at the beginning and end of the stimulation. Participants performed a perceptual learning task before and after stimulation. There were 2 sessions with stimulation frequency either at or above the resonance frequency of the primary somatosensory cortex (23 and 39 Hz, respectively). Perceptual learning occurred following above resonance stimulation only, while GABA reduced during this condition. Lower levels of early GABA were associated with greater perceptual learning. One possible mechanism underlying this finding is that cortical disinhibition “unmasks” lateral connections within the cortex to permit adaptation to the sensory environment. These results provide evidence in humans for a frequency-dependent inhibitory mechanism underlying learning and suggest a mechanism-based approach for optimizing neurostimulation frequency.

## Highlights

• In the context of repetitive sensory stimulation, GABA release is frequency dependent
• Stimulating above the resonance frequency of the somatosensory cortex reduces GABA
• Perceptual learning is associated with a reduction in GABA
• Early GABA reduction opens a window for plasticity and learning

## Graphical Abstract

**Figure:**
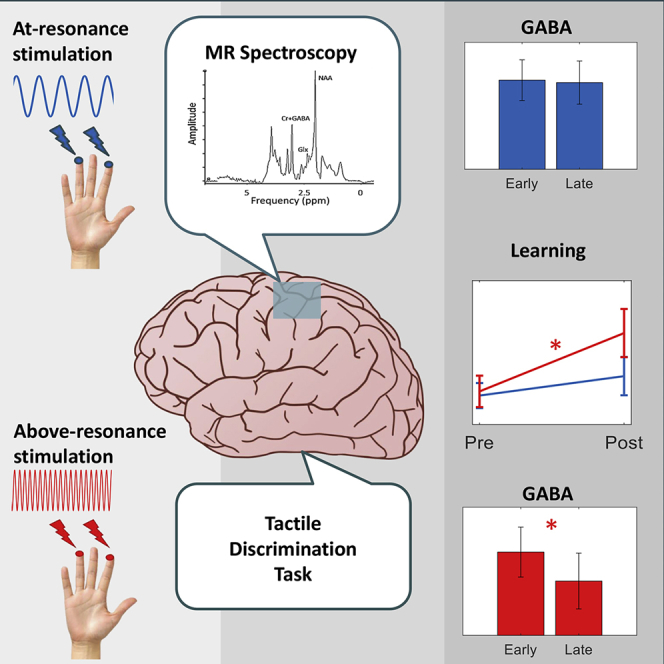

## Introduction

Plasticity is the process of altering synaptic efficacy via long-term potentiation (LTP) or long-term depression (LTD) which result in strengthening or weakening of the synapses, respectively (Malenka and Bear, 2004). A number of studies have indicated that the frequency of synaptic activation modulates plasticity at both excitatory and inhibitory synapses. At many glutamatergic synapses, high-frequency stimulation has been observed to induce LTP, whereas low-frequency stimulation promotes depression, a phenomenon known as frequency-dependent plasticity (FDP). FDP has been observed in various animal models (Bear and Abraham, 1996; Bliss and Gardner-Medwin, 1973; Bliss and Lømo, 1973; Kemp and Manahan-Vaughan, 2004; Kirkwood and Bear, 1994) with evidence that it may extend to sensory stimulation in humans (Lea-Carnall et al., 2017).

In humans, it has been shown that high-frequency tactile stimulation (20 Hz) delivered to the tip of the index finger improved 2-point tactile discrimination while low-frequency stimulation (1 Hz) impaired performance which was attributed to LTP-like changes in the primary somatosensory cortex (SI) (Ragert et al., 2008). It was noted in our recent work that 20 Hz is close to the stimulation frequency that evokes the maximum neural response of the somatosensory cortex, the resonance frequency (Snyder, 1992; Tobimatsu et al., 1999), and that the impaired performance at 1 Hz also applied to higher off-resonance frequencies (39 Hz) using tactile stimulation applied to two digits rather than one (Lea-Carnall et al., 2017), although this is likely via different mechanisms. Functional imaging showed that when the two digits were stimulated at the higher frequency (39 Hz), the digit representations in SI fused closer together which was associated with impaired performance on the tactile discrimination task, an observation interpreted by neural models as a result of strengthened connections between the regions. Conversely, when the two digits were stimulated close to the resonance frequency (23 Hz), performance was facilitated (as in (Ragert et al., 2008)) which was not accompanied by any shifting of the digit regions. Resonance is observed at every scale of cortical organization, from the individual synapse to complete brain regions across multiple animal models (Buzsáki et al., 2013; Hutcheon and Yarom, 2000; von Stein and Sarnthein, 2000). Cortical resonance is thought to be an emergent property of the network related to its features such as connection density, network size, and the biophysical properties of the constituent neurons (Hutcheon and Yarom, 2000; Lea-Carnall et al., 2016). It could be argued that such a property evolved in order to facilitate entrainment to the rhythmicity of the external environment (Hutcheon and Yarom, 2000).

Indeed, neural entrainment, the coupling of neural oscillators to an external stimulus, has been shown to be involved in a number of cognitive processes, regardless of sensory modality (Helfrich et al., 2019; Kösem et al., 2018; Obleser and Kayser, 2019; Pérez et al., 2019). Neural entrainment to the environment is crucial for structuring incoming information streams for further processing, including, attention selection, auditory sampling, and coupling neural rhythms to motor output (Lakatos et al., 2019). Entrainment is argued to be a basic mechanism that imparts temporal predictability and shapes sensory perception. Therefore, the selective neural and behavioral effects of entrainment at or away from the resonance frequency raise a number of questions about efficiency and adaptation of the brain's response to the environment. Understanding the mechanism of how or why stimulating at or off resonance results in different plasticity effects may help us to optimize stimulation paradigms in experimental and clinical settings.

There is growing evidence that the neurotransmitters gamma-aminobutyric acid (GABA) and glutamate (Glu) are functionally related to standard learning paradigms. For example, GABAergic disinhibition appears to be crucial to learning in the motor cortex (Floyer-Lea et al., 2006; Kolasinski et al., 2019; Stagg et al., 2011), and there appears to be a positive relationship between resting state Glu, glutamine (Gln), or GABA levels and plasticity in the visual and sensory cortices (Edden et al., 2009; Heba et al., 2016; Wijtenburg et al., 2017). In the case of perceptual learning, it has been shown that plasticity changes induced by presentation of repetitive stimulation applied to the finger tips is abolished in the presence of lorazepam, a GABA_*A*_ receptor agonist (Dinse et al., 2003a). A similar study found that application of memantine, an N-methyl-D-aspartate (NMDA) receptor blocker, completely eliminated plastic changes (and impaired performance) (Dinse et al., 2003b). A further study tested the effect of scopolamine, a cholinergic antagonist thought to inhibit NMDA-receptor activity (Falsafi et al., 2012), and found that too eliminated the effects of stimulation on plasticity compared to a control group (Bliem et al., 2008). Taken together, these results indicate a mechanistic role for neurotransmitters GABA and glutamate (which act on GABA and NMDA receptors, respectively) in perceptual learning.

Here, we tested the relationship between frequency-dependent plasticity and perceptual learning by co-stimulating two digits of the right hand at one of two frequencies: 23 Hz (at-resonance) and 39 Hz (above-resonance) while quantifying GABA_+_ (GABA + co-edited macromolecules) and Glx concentrations in contralateral SI using magnetic resonance spectroscopy (MRS). To test the consequence of the stimulation on perceptual learning, participants performed a forced-choice tactile discrimination test before and after stimulation. We predicted that performance on the tactile discrimination test would decline following above-resonance stimulation, in line with previous findings (Lea-Carnall et al., 2017). Furthermore, we predicted that GABA_+_ concentration would decrease during this condition only, supporting the idea that frequency-dependent perceptual learning is facilitated by frequency-dependent GABA_+_ reduction unmasking plasticity mechanisms. Additionally, we investigated the relationship between the change in GABA_+_ and Glx versus change in performance on the psychophysics test and we hypothesized, as in (Stagg et al., 2011), that we would find a correlation between the reduction in GABA_+_ and learning. Finally, we correlated GABA_+_ in the early phase of the experiment to overall changes in performance on the test and similarly we hypothesized that early levels of GABA+ might predict overall learning rates, as found in (Kolasinski et al., 2019).

## Results

During each visit, participants completed a forced-choice tactile discrimination test immediately before and after the repetitive stimulation. The test required subjects to locate the site of a brief vibrational pulse delivered to one of two fingers. Each participant was scanned twice and received either the at-resonance or above-resonance stimulation, in an order counter-balanced across participants. MRS data were acquired for the first (early) and last (late) 12 min of co-stimulation from a voxel within contralateral SI. The MRS voxel placement was determined in the axial plane as being centered on the “hand knob” area of the post-central gyrus and rotated in both the sagittal and coronal planes so that it was aligned to the cortical surface (Puts et al., 2011; Yousry et al., 1997), please see Methods for further details.

### Frequency-Dependent Perceptual Learning

Error rates for the forced-choice tactile discrimination tests were found to be 8.4 ± 5.5% prior to at-resonance stimulation (23 Hz) and 8.9 ± 6.9% prior to above-resonance stimulation (39 Hz). Intraclass correlation coefficient (ICC) verified the consistency of these mislocalization (ML) error rates over the two visits, see Equation (1). ICC(3,1) = 0.78 (p < 0.001, lower bound 0.45, upper bound 0.92). Post-stimulation ML error rates increased to 10.7 ± 8.5% after at-resonance and 15.8 ± 10.7% after the above-resonance co-stimulation.

Intersession reliability was assessed using ICC(3,1) (Shrout and Fleiss, 1979):

(Equation 1) ICC(3,1)=MSB−MSE/MSE+(k−1)∗MSE

where MSB is the between-subjects mean square, MSE is the error mean square, and k is the number of repeated sessions.

Linear mixed-model analysis revealed a main effect of discrimination test time (pre/post) (F = 12.6, p = 0.004), no main effect of stimulation frequency (at-resonance/above-resonance) (F = 2.8, p = 0.12), and discrimination test time by stimulation frequency interaction (F = 6.1, p = 0.03) on the error rate. Post-hoc linear regression of the difference in ML errors (post-pre) showed a significant increase of 77% (95% CI: 40%, 113%; p < 0.001) after above-resonance stimulation and no statistically significant effect of at-resonance stimulation with a change of 27% (95% CI: −12%, 67%; p = 0.16). That is to say, participants were more likely to confuse the site of a brief pulse delivered to one finger after 46 min of repetitive stimulation, but the increase was only significant after the above-resonance stimulation. We found no effects on reaction times.

### Frequency-Dependent Neurotransmitter Modulation

**Figure 1:**
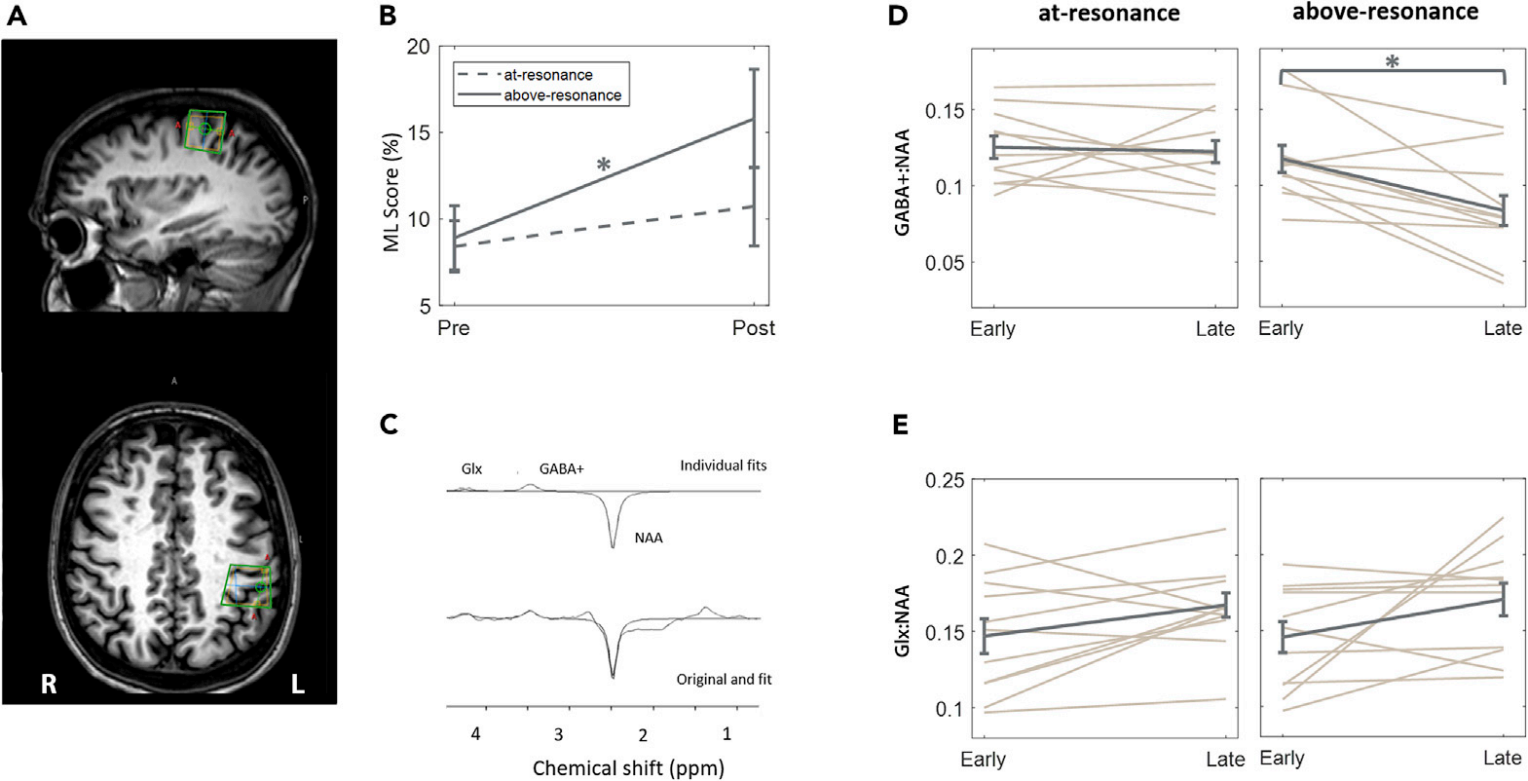
Frequency-Dependent Changes in Perception and Neurotransmitter Concentration (A) MRS was acquired from left SI using a voxel measuring 20 × 30 × 20 mm^3^. (B) Frequency-dependent perceptual learning: mean (and standard error) mislocalization rates on a forced-choice tactile discrimination task performed before and after 46 min of synchronous co-stimulation of D2 and D4 of the right hand using 23 Hz (at-resonance) and 39 Hz (above-resonance). Only above-resonance stimulation caused impaired performance on the task (F = 13.0, p = 0.003). (C) Example of an edited spectrum acquired with MEGA-PRESS and its fit by AMARES. The lower trace shows the original data and the fit, while the upper trace shows the components of the fit: NAA, Glx (Glu + Gln), and GABA_+_ (GABA + co-edited macromolecules). (D) Mean (and standard error) GABA_+_:NAA ratios acquired over the first and last 12 min of at-resonance stimulation (left) and above-resonance stimulation (right). A significant drop in GABA_+_ is observed during above-resonance stimulation only (F = 12.4, p = 0.005). (E) Mean (and standard error) Glx:NAA ratios acquired over the first and last 12 min of at-resonance stimulation (left) and above-resonance stimulation (right). No significant differences are observed.

GABA_+_ and Glx concentrations were estimated from averaged spectra over each of the two MRS blocks acquired during the first (early) and last (late) 12 min of the total 46 min of co-stimulation, see Figure 1D and 1E. Using N-acetylaspartate (NAA) concentration as a reference, we found GABA_+_:NAA ratio was reduced in response to above-resonance stimulation, coinciding with the impairment in performance on the discrimination test. There was no change in GABA_+_:NAA in response to at-resonance stimulation.

Linear mixed-model analysis on the GABA_+_:NAA ratio revealed a main effect of stimulation frequency (at-resonance/above-resonance) (F = 8.3, p = 0.02), a main effect for MRS block (early/late) (F = 8.8, p = 0.008), and an MRS block by stimulation frequency interaction (F = 5.4, p = 0.03). Post-hoc analysis of the differences between the MRS measurements (late-early) for the two stimulation frequencies showed a mean change in GABA_+_:NAA of −29% (95% CI: −45%, −13%; p = 0.001) for above-resonance stimulation and −3% (95% CI: −18%, 12%; p = 0.60) for at-resonance stimulation.

A similar test on the Glx:NAA concentrations found no effect of frequency (F = 0.03, p = 0.87), a main effect of MRS block (F = 5.9, p = 0.03), and no interaction (F = 0.15, p = 0.70). Post-hoc analysis of the differences (late-early) for each group showed a mean change in GLX:NAA of 17% (95% CI: −1%, 34%; p = 0.07) for above-resonance stimulation and 10% (95% CI: −7%, 29%; p = 0.23) for at-resonance stimulation.

We tested raw NAA in the same way as for the other metabolites and found no effect of stimulation frequency (F = 2.1, p = 0.18), no effect of MRS block (F = 3.3, p = 0.15), and no interaction (F = 2.5, p = 0.22). Finally, we tested the ratio of NAA:tCr and similarly found no effect of stimulation frequency (F = 0.3, p = 0.61), no effect of MRS block (F = 3.1, p = 0.12), and no interaction (F = 4.3, p = 0.09). A typical voxel placement is shown in Figure 1 (a) and an edited spectrum from a single participant is given in (c). We note here that spectral quality was consistent across the sessions, please see Table 1 for details of quality control metrics.

**Table 1:** The Quality Assessment Markers Are Reported Per Group

	At-Resonance		Above-Resonance	
	Early	Late	Early	Late
Subjects recruited	14	14	14	14
Subjects rejected	3	2	3	3
Subjects remaining	11	12	11	11
NAA LW	7.9 ± 1.7 Hz	8.1 ± 1.5 Hz	8.4 ± 3.2 Hz	7.9 ± 1.4 Hz
NAA CRLB (%)	5.3 ± 3.9%	5.4 ± 5.3%	9.4 ± 8.2%	8.4 ± 7.5%
GABA CRLB	1.6 × 10^−5^ ± 9.7×10^−6^	1.9 × 10^−5^ ± 9.2×10^−6^	1.4 × 10^−5^ ± 7.4×10^−6^	1.5 × 10^−5^ ± 6.2×10^−6^
GABA CRLB (%)	22.8 ± 5.8	24.8 ± 6.7%	24.7 ± 7.3%	41.0 ± 15.9%
SNR	13 ± 2.5	13 ± 3.4	15 ± 4.3	14 ± 5.3

LW: linewidth; CRLB: Cramer-Rao lower bounds, which is presented as an absolute value for GABA+ and as a percentage of amplitude for both GABA+ and NAA; SNR: time-domain signal-to-noise ratio. Values are presented as mean ± standard deviation (SD) for the final list of included datasets. Linear mixed-model analysis on the NAA LW and SNR showed no effects of the stimulation.

### Relationship between GABA, Glx, and Perceptual Learning

**Figure 2:**
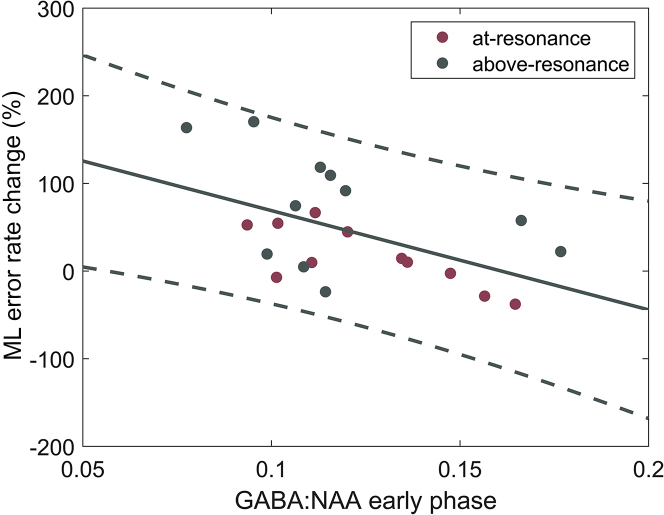
GABA_+_ Concentration at Onset of Stimulation Is Correlated with Perceptual Learning Outcomes GABA_+_:NAA concentrations in the early MRS block are plotted against the percentage difference in ML score, a measure of perceptual learning, for each participant for each of the stimulation conditions: at-resonance (red), above-resonance (green). We find a negative correlation (R = −0.51, p = 0.01) across both conditions which was maintained for the at-resonance condition (R = −0.75, p = 0.008). 95% CI (all data) shown by dotted lines.

Initially, we tested the relationship between GABA_+_:NAA quantified from the first 12 min of the stimulation with the percentage difference in performance on the ML task (pre to post stimulation) over both conditions combined using Pearson correlation analysis. There was a significant negative correlation between GABA_+_:NAA at the start of stimulation and change in ML error rate (R = −0.51, p = 0.01) which was maintained for the at-resonance condition (R = −0.75, p = 0.008) but not for the above-resonance condition (R = −0.42, p = 0.19), see Figure 2. In other words, subjects who had lower levels of GABA_+_:NAA at the beginning of the stimulation went on to have greater increases in their ML error rates post stimulation. We interpret impairment of task performance as being indicative of plastic changes in SI, as in (Pilz et al., 2004).

Next, we tested whether the percentage difference in GABA_+_:NAA between the early and late MRS blocks was correlated with the percentage difference in the ML score and found no correlation (R = 0.09, p = 0.70).

These tests were repeated for Glx:NAA and no correlation with learning was found, neither in the early MRS block (R = −0.04, p = 0.88), nor the percentage difference for GLX:NAA between the early and late MRS blocks (R = 0.28, p = 0.21).

## Discussion

Evidence from physiological recordings has shown that when endogenous brain oscillations phase align to salient events in a sensory stream, those events are processed and perceived more readily than non-aligned events (Schroeder and Lakatos, 2008). In other words, and regardless of the sensory modality, neural entrainment to the environment is crucial for structuring incoming information streams for further processing (Besle et al., 2011; Helfrich et al., 2019; Lakatos et al., 2019). Neural entrainment imparts temporal predictability that is argued to be a basic mechanism that shapes sensory perception. However, the neural and cognitive processes that arise due to entrainment seem to be frequency dependent (Lea-Carnall et al., 2017; Ragert et al., 2008). Of particular interest is how the frequency of the entraining stimulus selectively enables plasticity mechanisms and the special role of GABA in modulating such frequency-dependent plasticity (Davies et al., 1991).

Here, we combined psychophysics testing with MR spectroscopy to investigate the neuromodulatory basis of frequency-dependent plasticity and its role in perceptual learning. We used a paradigm of digit co-stimulation (Pilz et al., 2004; Vidyasagar et al., 2014) to induce FDP in human SI at two different frequencies. We confirmed frequency specific changes in perception as previously reported (Lea-Carnall et al., 2017). Subjects were more likely to mislocalize a brief pulse delivered to one finger following above-resonance stimulation which is interpreted as an indicator of cortical reorganization (Pilz et al., 2004). We quantified GABA_+_ and Glx during the first and last 12 min of the co-stimulation to test for frequency-specific changes of these metabolites and whether such changes related to FDP and perceptual learning. Using NAA concentration as a reference, we found that GABA_+_:NAA ratio was frequency dependent, in that only above-resonance stimulation reduced this ratio during the course of the experiment. No changes in Glx:NAA were found. Further, we found that the magnitude of change in ML errors correlated with the concentration of GABA_+_:NAA during the first 12 min of co-stimulation which may be an indication that early disinhibition of the GABAergic systems is important to learning as has been found in the motor cortex (Kolasinski et al., 2019).

Variants of the digit co-stimulation paradigm have been used to study plasticity. Synchronous co-stimulation of multiple points on a single digit (or paw) has been shown to improve 2-point tactile discrimination in humans and animals, accompanied by topographical changes in SI evidenced by electroencephalography (EEG), magnetoencephalography (MEG), and functional magnetic resonance imaging (fMRI) (Godde et al., 2003, 1996; 2000; Hodzic et al., 2004; Pleger et al., 2001). Synchronous co-stimulation of multiple digits has been shown to impair 2-digit discrimination and fused or reduced the distance between the cortical representations of digit regions as measured by fMRI, whereas asynchronous stimulation has the opposing effect (Pilz et al., 2004; Vidyasagar et al., 2014). In this study, we tested the effect of stimulation frequency on plasticity outcomes and found that above-resonance stimulation led to worsening of performance on a tactile discrimination test, whereas at-resonance stimulation did not, in agreement with previous findings (Lea-Carnall et al., 2017). We interpret a worsening error rate after stimulation as being the result of increased lateral connectivity between these regions due to the stimulation (Pilz et al., 2004).

It has been suggested that the mechanisms underlying perceptual learning are the same as those underlying LTP in general which rely on NMDA receptor activation (Dinse et al., 2003b). This is supported by pharmacological studies by Dinse et al. who showed that lorazepam, a GABA_*A*_ agonist, completely eliminated the effects of a digit stimulation protocol known to induce plasticity (Dinse et al., 2003a). We observed a reduction in GABA_+_ concentration in the above-resonance condition only, which was associated with impaired performance on the behavioral task. fMRI has shown that above-resonance stimulation causes the digit regions to shift closer together and functional connectivity between them to increase, supported by modeling work indicating that this is commensurate with a strengthening of lateral connections (Lea-Carnall et al., 2017). Lower levels of tonic GABA may facilitate this process. We suggest that by strengthening connections between the digit representations within SI, their local activation maxima broaden and overlap on the cortical surface. An incoming stimulus is then more likely to activate an area within the overlapping region leading to increased ML errors on the task (Pilz et al., 2004; Vidyasagar et al., 2014). More generally, these results are in line with findings from other authors that have observed an association between a reduction in GABA concentration in the relevant brain regions and LTP-like plasticity in animals (Wigstrom and Gustafsson, 1983; Davies et al., 1991), and both healthy (Floyer-Lea et al., 2006; Kolasinski et al., 2019; Stagg et al., 2011) and clinical human populations (Levy et al., 2002; Blicher et al., 2014). With regard to FDP specifically, early animal studies have indicated that inhibitory GABAergic autoreceptors may underlie the phenomenon of FDP (Davies et al., 1991). At low frequencies, excitatory potentiation is inhibited by GABAergic neurons, whereas, high-frequency stimulation causes disinhibition of this system allowing excitatory potentiation to occur. Our results suggest that a similar mechanism may also be present here.

We report a negative correlation between SI GABA_+_:NAA in the early MRS block and overall magnitude of perceptual learning across both conditions, with lower early levels of GABA_+_:NAA relating to higher levels of impairment on the tactile discrimination test. This correlation persisted for the at-resonance condition but not for the above-resonance condition, which was surprising given the latter condition facilitated the greatest levels of learning as measured by the ML test. In a recent study, Kolasinski et al. similarly showed that GABA concentration acquired in M1 in the early phase of a motor learning task negatively correlated with the overall degree of learning but this effect was not present in a control condition (Kolasinski et al., 2019). We found no evidence of learning for the at-resonance condition at the group level. However, we cannot rule out learning at the level of each individual. As the 23 Hz stimulation was fixed for all participants and was not calibrated individually, it is likely that it was just below or above the actual resonance frequency for each participant. The resonance frequency of SI is thought to be between 20 and 26 Hz (Snyder, 1992; Tobimatsu et al., 1999), meaning there may have been considerable variability between the participants and this may be driving any differences in their response to the stimulation. If early GABA levels are predictive of overall learning, then it may be the case that there is a certain “critical threshold” level for GABA within a neuronal circuit which once breached triggers plasticity cascades which affect further inhibitory processes in a nonlinear way. If this is the case, then the correlation between early GABA_+_:NAA and learning in the above-resonance condition might be lost. The general finding, however, that early levels of GABA predict learning and performance fits with our current understanding of the role of GABA in sensory and motor systems. Across different sensory modalities, higher baseline GABA levels are positively associated with enhanced sensory function and acuity (Kolasinski et al., 2017; Edden et al., 2009), as well as induced plasticity changes as assessed by task performance (Heba et al., 2016). Taken together, these results indicate a potential role for early frequency-dependent cortical disinhibition in perceptual learning.

We did not observe a direct correlation between the late minus early change in SI GABA_+_:NAA and the equivalent change in ML scores. Other studies have shown a relationship between alterations in GABA concentration and plasticity in the motor cortex (Floyer-Lea et al., 2006; Stagg et al., 2011). However, an important difference is that our MRS acquisition started concurrently with the stimulus providing an insight into ongoing GABA processes, whereas in the above studies, GABA concentrations were acquired before and after an intervention. It may also be that the relationship between the change in GABA_+_:NAA and change in task performance is nonlinear which our correlation analysis would fail to detect. Finally, we note that the physiological basis for a functional change in GABA concentration is unclear (Stagg et al., 2011) and may be related to a change in GABA metabolism or a relative shift of GABA to a state/compartment that is less “visible” to the MR scanner, for example, due to shortened T2∗ (Mullins, 2018). Altered GABA_+_:NAA between the early and late blocks may be due to different physiological mechanisms and may therefore relate to plasticity (ML measures) differently.

In a previous study, we found that at-resonance stimulation sped up reaction times (Lea-Carnall et al., 2017); this effect was absent here. While the protocol was the same, the experimental environment differed between the two studies. In (Lea-Carnall et al., 2017), psychophysics data were collected outside the scanner at 20-min intervals over 60 min of co-stimulation and the ML tests were performed immediately after co-stimulation. In the current study, the stimulus was applied while the subjects were in the scanner for 46 min, experiencing concurrent repetitive mechanical and acoustic stimuli, almost certainly off-resonance, which may have interfered with the 23 Hz stimulus. Further, upon completion of the stimulation, there was a 10-min period within which the participants completed an fMRI task that also involved vibrational pulses delivered to the digits and only after that they were removed from the scan room and asked to perform the final ML test.

We do not report any change in Glx:NAA during either stimulation condition. Glx is a composite measure of the combined concentration of Glu and Gln reported due to the difficulty of Glu quantification with AMARES using the GABA-edited MEGA-PRESS sequence which does not generally allow for the separation of these resonance peaks in the spectrum. With regard to the specific role of Glu in plasticity, it is well established that NMDAR activity mediates LTP allowing potentiation of the presynaptic signal via increased receptor trafficking. However, MRS is sensitive to glutamate concentrations rather than measures of receptor density. There is evidence that Glu presynaptic release rates might increase as a result of both Hebbian and homeostatic plasticity, but this is dependent on a number of factors (see (Costa et al., 2017) for a review). It has been shown that by blocking NMDAR activity using memantine (Dinse et al., 2003b) or scopolamine (Bliem et al., 2008), the effects of co-stimulation on perceptual learning are abolished, indicating that NMDARs play a critical role in this type of learning. Glu is abundant in the human brain and is involved in a plethora of neural processes (Ramadan et al., 2013). It may be that changes in glutamate in relation to plasticity are small in magnitude compared to global glutamate levels, and therefore, teasing out the specific effect attributable to plasticity is difficult.

While entrainment to sensory stimulation has been shown to have behavioral and neural consequences (Lakatos et al., 2019), similar results have been achieved using exogenous transcranial alternating current (tACS) stimulation (see (Herrmann et al., 2013; Thut et al., 2011), for reviews). In a recent study, Nowak et al. applied beta (20 Hz, at-resonance) and gamma (75 Hz, above-resonance) tACS stimulation over primary motor cortex (Nowak et al., 2017). They found a duration-dependent reduction in resting state GABA_*A*_ inhibition, quantified by short interval intracortical inhibition, following gamma stimulation only which was not present following beta stimulation. These results help corroborate the observation that the triggering of GABAergic mechanisms coincided with above-resonance stimulation, although we acknowledge that the relationship between physiological measurements of inhibition and MRS GABA is unclear (Dyke et al., 2017; Stagg et al., 2011). On the other hand, Vossen et al. attributed the persistence of occipital alpha oscillations following individualized alpha tACS to plasticity, even though the alpha is or is close to the resonance frequency of the occipital cortex (Vossen et al., 2015). Similarly, beta tACS entrainment (at-resonance) has been shown to induce NMDAR-mediated plasticity in the human motor cortex using pharmacological intervention (Wischnewski et al., 2019). However, gamma tACS entrainment of the motor cortex (above-resonance) appears to have the unique effect of boosting plasticity via GABA_*A*_ disinhibition as has been replicated in the literature (Guerra et al., 2019, 2018). It has been suggested that tACS targets interneurons and pyramidal cells differently, affecting the overall balance of activity and furthermore that inhibitory subnetworks which exhibit resonance frequencies in the gamma range may be more susceptible to activation by similar stimulus frequencies (Nowak et al., 2017; Wischnewski et al., 2019). While we are cautious about drawing comparisons between these two stimulus types (tACS and sensory stimulation), we suggest that it is plausible that entrainment via either method using above-resonance frequencies has a similar mechanism of action on inhibitory circuits.

The scan session in this study was particularly long (approx. 70 mins), and therefore, it is expected that participants experienced a decline in attention by the end which may have had an effect on ML test performance. It is generally acknowledged that attention plays a crucial role in functional activation and learning (Ahissar and Hochstein, 1993; Buchner et al., 1999; Recanzone et al., 1992). However, studies have consistently shown that perceptual learning in response to repetitive sensory stimulation (even below detection thresholds) occurs even in the absence of attention in both the visual (Watanabe et al., 2002) and sensory systems (Dinse et al., 2003a; Godde et al., 2003, 2000; Heba et al., 2016; Hodzic et al., 2004; Lea-Carnall et al., 2017; Pleger et al., 2001, 2003). If GABA disinhibition is required for the induction of perceptual learning, then we would expect to observe the changes in GABA_+_:NAA concentration in this experiment regardless of whether participants attended to the stimulus or not. We also note that participants were reminded before completing each test of the importance of performing to the best of their ability maintaining their integrity as scientific research subjects. If lethargy and lack of attention played a role in the diminished ML scores, then we expect this to be balanced across the conditions.

In a previous study, we showed that at-resonance stimulation consolidates a network, whereas above-resonance recruits a broader network. While the determination of the precise relationship between at/above resonance stimulation and performance of different cognitive tasks remains an open question, the results suggest that in applications where plasticity is of interest, for example, for learning novel environments, entraining at a frequency away from resonance of the target network may be advantageous. Identifying the preferred or resonance frequency of a network, nested within other networks, may not be straightforward but could be determined experimentally and would depend on the context and sensory and cognitive content of the stimuli (Besle et al., 2011; Ding et al., 2006; Lea-Carnall et al., 2016).

In conclusion, our findings suggest that frequency-dependent early changes in inhibitory signaling are correlated with perceptual learning outcomes. Furthermore, we bridge a gap between cellular and systems levels of investigation frequency-dependent plasticity and highlight the importance of distinguishing between at-resonance and off-resonance frequencies of a particular network. From a practical viewpoint, our results suggest that repetitive sensory stimulation could selectively switch plasticity mechanisms by modulating inhibitory processes GABA. This could prove useful in optimizing stimulation strategies to suit different rehabilitation or cognitive enhancement purposes.

### Limitations of the Study

In this study, we compared SI MRS GABA_+_:NAA measured during the first and last 12 min of co-stimulation of the digits. For a complete picture of GABA dynamics, a baseline measurement taken before the onset of stimulation would be optimal. In addition, MRS data were not corrected for GM/WM variations in the tissue, making this a potential confound. However, since the MRS voxel was not re-positioned between the early and late acquisitions, this cannot contribute to the changes seen between the early and late MRS blocks. Our experimental design ensures that any variation in GM/WM ratio or macromolecule concentration due to differing voxel placement between subjects and sessions was unlikely to influence the findings. The “at” and “above” resonance stimulation conditions were performed in a randomization order between participants, and the radiographer placing the voxel was blinded to the condition, meaning there could be no bias in voxel placement between conditions. Finally, the duration of the scan as well as acoustic and mechanical vibrations from the scanner might have interfered with the tactile stimulation, causing fatigue and dampening the frequency-dependent plasticity effect.

### Resource Availability

#### Lead Contact

Further information and requests for resources should be directed to and will be fulfilled by the Lead Contact, Caroline Lea-Carnall (caroline.lea-carnall@manchester.ac.uk).

#### Materials Availability

This study did not generate new unique reagents.

#### Data and Code Availability

The data collected in this study are publicly available at https://doi.org/10.6084/m9.figshare.13026320.

## Methods

All methods can be found in the accompanying Transparent Methods supplemental file.

## Supplemental Information

Document S1. Transparent Methods and Figure S1
