# Supplementary material for: GABA Modulates Frequency-Dependent Plasticity in Humans
Source: iScience. 2020 Oct 8;23(11):101657. doi: 10.1016/j.isci.2020.101657 (PMC7599432; doi:10.1016/j.isci.2020.101657)
Supplement: Document S1. Transparent Methods and Figure S1 [file mmc1.pdf]

iScience, Volume *23*

Supplemental Information

GABA Modulates

Frequency-Dependent

Plasticity in Humans

Caroline A. Lea-Carnall, Stephen R. Williams, Faezeh Sanaei-Nezhad, Nelson J. Trujillo-Barreto, Marcelo A. Montemurro, Wael El-Deredy, and Laura M. Parkes

Supplementary Information

1 Transparent Methods

1.1 Subjects and ethics statement

This study was approved by the University of Manchester Research Ethics Committee (UREC Ref 2018-4395-6120) and fully informed written consent was obtained from all subjects prior to participation. Fourteen healthy right-handed subjects (7 male, age range 20-49 years old) were scanned twice using a 3 T Philips Achieva MRI with an 8-channel head coil.

1.2 Experimental protocol

A schematic design of the study can be seen in Fig. S1 which indicates the temporal order of the scans and tests used in the study. MRS data was collected during tactile co-stimulation at 2 different frequencies in order to measure changes in GABA_{+} and Glx. A tactile discrimination task was performed before and after scanning to measure the effect of co-stimulation on tactile perception with worsening scores assumed to be an indicator of plastic change (Pilz et al., 2004). Participants first completed the tactile discrimination test while seated in a quiet room near to the scanner in order to measure their baseline ability. They were then immediately positioned in the scanner and vibrotactile stimulators were placed in the middle of the fleshy pads of digits 2 and 4 (the index and ring fingers) of the right hand and secured with tape. A T_1-weighted image with 1 mm isotropic resolution was acquired followed by fMRI (not reported here). There followed a period of 46 minutes of simultaneous in-phase tactile co-stimulation of digits 2 and 4 using either at-resonance (23 Hz) or above-resonance (39 Hz) frequency stimulation in order to bring about plastic change as reported in our earlier study (Lea-Carnall et al., 2017). MRS data was acquired for the first (early) and last (late) 12 minutes of co-stimulation in order to measure any changes in GABA_{+} or Glx due to the ongoing application of the co-stimulation. Resting state fMRI was collected in the gap between MRS. Upon completion of scanning, participants were removed from the scanner and immediately moved to the nearby testing room to repeat the tactile discrimination test. The tactile stimulators consist of a ceramic box containing a vibrating probe driven by a piezoceramic wafer (Dancer Designs Ltd) which were controlled using Matlab software (http://www.mathworks.com). Each digit had its own stimulator which was driven with the correct vibrational frequency delivered in-phase to each digit with un-loaded peak to peak amplitude of 250 m. Each participant was scanned twice at least 2 weeks apart and received either the at-resonance or above-resonance stimulation, in an order counter-balanced across participants.

1.3 Tactile discrimination test

Participants were seated and the vibrotactile stimulators were placed in the middle of the fleshy pads of digits 2 and 4 of the right hand and secured with tape. Short pulses (500 ms) were delivered randomly to one of the two digits, at one of three intensity levels, and participants were asked to decide which digit they felt the pulse

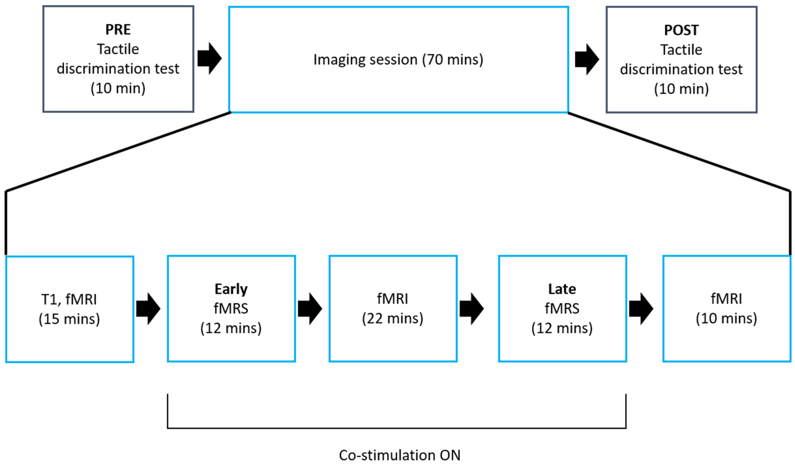

Figure S1: Schematic design of the study. Related to Figure 1.
Schematic of one (of two) scans of the experimental protocol. Initially, participants completed a forced-choice tactile discrimination test outside the scanner. The test required them to detect which finger received a short vibrational pulse (500 ms). Once inside the scanner, a high-resolution T1-weighted image was acquired to guide the placement of the spectroscopy voxel. MRS was collected during the first and last 12 mins of 46 mins total of co-stimulation at one of the two frequencies (at or above resonance). Once participants left the scanner, they immediately repeated the forced-choice tactile discrimination test. fMRI was also collected and that is reported elsewhere Lea-Carnall et al. (2017). The scan session and testing was repeated using the other stimulation frequency a minimum of two weeks later.

in. The pulse intensities determined detection difficulty and were defined in terms of the displacement distance of the probe (Easy 8.3 m; Medium 5.6 m; and Hard 2.8 m) above the average detection threshold calculated from data collected previously (Lea-Carnall et al., 2017) (mean 16.1 m, SD 4 m, calculated for both digits from 15 participants with a similar age and gender distribution to the participants in this study). The test was programmed using Presentation (Neurobehavioural Systems, www.neurobs.com). A total of 180 pulses were delivered in a random order with an inter-trial interval of 2s. Responses were recorded with the left hand using a keyboard. The tactile mislocalisation rate is defined as the percentage of times a participant incorrectly classified the stimulus as belong to a specific digit (non-responses allocated at 50% accuracy). The stimulus was applied at a range of difficulty levels in order to try to capture an intensity just above the participant's true detection threshold given that we used an estimated value for this experimental parameter. For this reason, mislocalisation test results are reported as means averaged across difficulty levels for each participant. Reaction times were also recorded.

1.4 Data acquisition

GABA-edited spectra were acquired using a J-difference-edited MEshcher–GArwood Point RESolved Spectroscopy (MEGA-PRESS) sequence (Mescher et al., 1998) from participants' left SI with a voxel size of 20 $\times$ 30 $\times$ 20 mm^3 for the first and last 12 minutes of the tactile stimulation. A 1 mm isotropic T1-weighted image was acquired to aid the positioning of the 1H MRS voxel. A typical voxel placement is shown in Fig. 1 (a). The MRS voxel placement within SI was determined in the axial plane as being centred on the "hand knob" area of

the post-central gyrus and rotated in both the sagittal and coronal planes so that it was aligned to the cortical surface (Puts et al., 2011; Yousry et al., 1997). The MRS voxel was placed at the beginning of the scan session and was not moved between the early and late MRS blocks. Placement of the MRS voxel was replicated between experimental conditions (the 2 scan sessions relating to the 2 stimulation frequencies) by eye by referring to images of the initial placement in 3 planes by a radiographer who was blinded to the conditions and was then checked by another researcher. Great care was taken to minimise error and as the conditions were performed in a randomised order between participants, there was no bias for positioning between the conditions or movement of the participant between early and late MRS blocks.

In the initial phase of the study (10 subjects), the spectra were acquired in blocks of 4 acquisitions when the MEGA pulse was set at 1.89 ppm (MEGA-on), referred to as a single dynamic, followed by 4 acquisitions of MEGA pulse set at 7.6 ppm (MEGA-off) There were 90 dynamics in total (45 ON and 45 OFF). For the final 4 subjects, a spectrometer upgrade enabled us to interleave single spectra of MEGA-on and MEGA-off off with 352 spectra in total (176 ON and 176 OFF). The dynamics were then repeated in an interleaved manner for 12 mins. Echo time (TE) was 70 ms, repetition time (TR) was 2000 ms with 1024 samples. Receiver bandwidth was 2000 Hz, water suppression method was excitation with a window of 140 Hz and the shimming was second order pencil beam. MRS acquisitions followed EPI and whilst it is known that EPI can induce frequency instability in MRS acquisitions, we overcame any effects of this by frequency aligning dynamics before calculating the edited spectra. It should also be noted that any effects of EPI in the data would be present in both conditions. Using data from power calculations given in (Sanaei Nezhad et al., 2020) we used a sample size of 14 which powered our data to detect a 20$\%$ change in $GABA_+$ in SI (using the motor cortex as a reference).

1.5 Metabolite quantification

The acquired MEGA-PRESS spectra were exported from the scanner as single time-domain free induction decays for processing using jMRUI v6.0 (Naressi et al., 2001; Stefan et al., 2009). The spectra were aligned using the 'automatic alignment' option in the jMRUI pre-processing menu and were then summed (effectively subtracting the MEGA-OFF from the MEGA-ON spectrum due to the 180 degree phase shift in the MEGA-OFF acquisition). The resultant edited spectra was phase-corrected automatically by jMRUI using the co-acquired non-water suppressed acquisition. The N-acetylaspartate (NAA) peak at 2.02 ppm was used for frequency referencing.

$GABA_+$, conventionally taken to mean GABA plus co-edited macromolecules (Mullins et al., 2014) was estimated from the edited spectrum using the AMARES routine (Mierisova et al., 1998), which fits single resonances in the time-domain using frequency and line shape prior knowledge. AMARES analysis also provides a measurement of glutamate + glutamine (conventionally abbreviated as Glx). Data are presented as ratios to NAA in the MEGA-edited spectrum as this provides an internal correction for any variations in editing efficiency.

1.6 Statistical analysis

Tactile mislocalisation rates for each participant were averaged across the three difficulty levels for each testing session. Initially, we evaluated intersession reliability of the mislocalisation baseline (comparing pre condition measured before scanning and repeated at least two weeks apart for each participant) measurement using the ICC (please see Equation 1 for definition).

Due to missing values for some of the subjects, linear mixed model analyses were used to assess the main effect of MRS Block (early/late - relating to the first and last 12 mins of co-stimulation) and Stimulation Frequency (at-resonance/above-resonance), and an interaction between MRS Block and Stimulation Frequency on the $GABA_+$

and Glx levels. We conducted a similar test to assess the main effects of Mislocalisation Test Time (pre/post) and Stimulation Frequency (at-resonance/above-resonance), and an interaction between Mislocalisation Test Time and Stimulation Frequency on the participants' error rates and reaction times. Finally we tested the reference metabolite NAA as well as the ratio NAA:tCr (creatine + phosphocreatine) in the same way.

Normality was assessed using the Wilk-Shapiro test on the residuals, and Mauchly's sphericity test was conducted to examine whether the assumption of sphericity had been violated. No violations were found in the present study.

Finally, we assessed whether levels of SI $GABA_{+}$:NAA and Glx:NAA in the early MRS block or the magnitude of its change across the early and late acquisitions predicted subsequent learning using Pearson correlation analyses. We calculated correlations for each condition separately and combined. All statistics were computed in R (R Core Team, 2013).

1.7 Quality control and exclusion criteria

Edited MEGA-PRESS spectra were initially inspected by eye to identify any of poor quality to be excluded from analysis. A number of spectra were seen to be noisy so signal-to-noise ratio (SNR) in the time-domain was calculated for all spectra using AMARES. Spectra were excluded if SNR < (mean SNR - minus two standard deviations (SD)), in which the mean was calculated without excluded spectra. Practically this involved rejecting spectra which fell outside 2 SD below the global mean, then recalculating the mean and SD excluding the rejected spectra and iteratively removing new outliers. 11/72 spectra were rejected and a table of quality control metrics per group is given in Table 1.

References

Lea-Carnall, C., Trujillo-Barreto, N., Montemurro, M., El-Deredy, W. and Parkes, L. (2017), 'Evidence for frequency-dependent cortical plasticity in the human brain', *Proc Natl Acad Sci U S A* **114**(33), 8871–8876.

Mescher, M., Merkle, H., Kirsch, J., Garwood, M. and Gruetter, R. (1998), 'Simultaneous in vivo spectral editing and water suppression', *NMR Biomed* **11**(6), 266–272.

Mierisova, S., van den Boogaart, A., Tkac, I., Van Hecke, P., Vanhamme, L. and Liptaj, T. (1998), 'New approach for quantitation of short echo time in vivo 1h mr spectra of brain using amares', *NMR Biomed* **11**, 32–39.

Mullins, P., McGonigle, D., O'Gorman, R., Puts, N., Vidyasagar, R., Evans, C., on MRS of GABA, C. S. and Edden, R. (2014), 'Current practice in the use of mega-press spectroscopy for the detection of gaba', *Neuroimage* **1**(86), 43–52.

Naressi, A., Couturier, C., Castang, I., de Beer, R. and Graveron-Demilly, D. (2001), 'Java-based graphical user interface for mrui, a software package for quantitation of in vivo/medical magnetic resonance spectroscopy signals', *Comput Biol Med* **31**, 269–286.

Pilz, K., Veit, R., Braun, B. and Godde, G. (2004), 'Effects of co-activation on cortical organization and discrimination performance', *Neuroreport* **15**, 2669–2672.

Puts, N., Edden, R., Evans, C., McGlone, F. and McGonigle, D. (2011), 'Regionally specific human gaba concentration correlates with tactile discrimination thresholds', *J Neurosci* **31**(46), 16556:16560.

R Core Team (2013), *R: A language and environment for statistical computing.*, R Foundation for Statistical Computing, Vienna, Austria.

Sanaei Nezhad, F., Lea-Carnall, C., Anton, A., Jung, J., Michou, E., Williams, S. and Parkes, L. (2020), 'Number of subjects required in common study designs for functional gaba magnetic resonance spectroscopy in the human brain at 3 tesla', *Eur J Neurosci* **51**(8), 1784–1793.

Stefan, D., Di Cesare, F., Andrasescu, A., Popa, E., Lazariev, A., Vescovo, E., Strbak, O., Williams, S., Starcuk, Z., Cabanas, M., van Ormondt, D. and Graveron-Demilly, D. (2009), 'Quantitation of magnetic resonance spectroscopy signals: the jmrui software package', *Measurement Science and Technology* **20**(10), 104035–104044.

Yousry, T., Schmid, U., Alkadhi, H., Schmidt, D., Peraud, A., Buettner, A. and Winkler, P. (1997), 'Localization of the motor hand area to a knob on the precentral gyrus: a new landmark', *Brain* **120**(1), 141–157.
